# Book Review

**Published:** 1912-09

**Authors:** 


**The Etiology, Diagnosis and Prophylaxis of Pulmonary Phthisis.** By ALFRED HARRIS, M.B., Ch.B. Vict., D.P.H. Camb. Pp. 126. Bristol: John Wright & Sons Ltd. 1912.—This short treatise of 120 pages is somewhat slight for its comprehensive title. The historical survey in Section I and the following section on etiology contain many interesting facts in the form of references and quotations. The best part of the work is in Section IV on the "exciting cause," which deals with the morphology and other characteristics of the tubercle bacillus. The author makes the unqualified statement that "tubercle bacilli are not found in the urine of cases of phthisis, unless the bladder or other portion of the genito-urinary tract is affected." There is no doubt that bacilli can be found in the urine of many cases of phthisis which present no evidence of *clinical* genito-urinary tuberculosis. Flick and Walsh go so far as to say that fifty per cent. of phthisics have tuberculosis of the kidney, and that bacilli can be found in the urine of *all* cases of active pulmonary tubercle. Section VII. on prophylactic treatment is good. Attention is called to the danger from flies, and some interesting experiments are detailed. The appendices on sanatorium treatment and tuberculin treatment are too sketchy to be of much use. The writer is evidently well versed in the literature and history of tuberculosis, but the work contains little that is new, and we feel that the author would have made better use of his material had he employed it as the basis of some more ambitious work on the lines of a text-book.

**On Bronchial Asthma: its Pathology and Treatment.** By J. B. BERKHART, M.D. Revised and abridged Third Edition. Pp. vi, 150. London: Oxford University Press. 1911.—Seldom have we read a medical monograph with the sense of disappointment that the one before us has afforded. Not until we reach p. 80 is the author's hypothesis as to the actual cause of the asthmatic attack revealed, viz. that asthma is a symptom of fibrinous bronchitis, of which latter a micro-organism is the cause, yet but scanty evidence in support of this view is advanced. The author seems more concerned to use hard terms than sound argument against the more prevalent theories. "To shake the sacred edifice of a bronchial spasm would naturally rouse the anger of the devout votaries at that shrine." "It is unnecessary to refer to a diagnosis of the alleged bronchial spasm . . . the attendant expiratory dyspnœa cannot be due to a bronchial spasm, as such hypothetical process would impede inspiration but facilitate expiration." One might forgive summary dismissal of widely-accepted theories if only the author gave us some grounds for throwing them aside and scientific argument in favour of his own, but he tells us "it is unnecessary at present to defend the view which I had adopted. A host of observers have since then declared in favour of the bacterial origin of acute bronchitis." The section on treatment is equally unsatisfying.

**Studies in Pulmonary Tuberculosis.** By Frederick Guy Griffiths, M.D. Pp. iii, 113. London: Baillière, Tindall & Cox. 1911.—This work is an amplification of the prize thesis for the degree of Doctor of Medicine in the University of Sydney, and is founded on work done under the teaching of Dr. Camac Wilkinson. Chapter vii, on the specific treatment of pulmonary tuberculosis, speaks of the original method of Koch, elaborated by Wilkinson, which begins with small doses and progresses to larger ones, until the dose may become 60,000 times as large as the original dose; and Wilkinson maintains that it is only by enormous doses like these that one can hope to produce permanent effects. This book gives much evidence on the one side of the story, but the limitations of a potent drug like tuberculin are obvious, and it should be pushed to full doses only with caution and under close observation by one of experience and knowledge. We remember what harm was done by tuberculin given rashly in the early days, and this remembrance is now bearing fruit in a degree of caution, which is sometimes perhaps over-strained.

**Consumption: Treatment at Home and Rules for Living.** By H. Warren Crowe, M.D. Pp. 36. Bristol: John Wright & Sons Ltd. 1912.—This third edition should be more useful than ever. In thirty-six pages the author gives a series of rules for self-management, which should be helpful to all whom it may concern. Rule 23 reads as follows: "At each meal eat as much as ever you can, then drink a pint of milk." This is a sample. The practice may not always be possible.

**Health to Date.** By W. T. Fernie, M.D. Pp. xvi, 477. Bristol: John Wright & Sons Ltd. 1911.—Dr. Fernie in his preface speaks of this as his "Swan-Song," but we trust that in spite of the advanced years to which he confesses this may not prove his last venture in print. The sub-title, "The Modern Doctor; with Newer Methods of Cure," discloses the scope of the volume. All our new theories and plans of treatment come under the kindly-humorous criticism of Dr. Fernie; and strange enough is the figure cut by some of our modern fashions in physic, when the author out of the fulness of his experience and the rich store of his information parallels them with some ages-old crank or fad. Discursive, indeed, is his method, but always to some purpose. Old customs and apt quotations both old and new come in pat for illustration. "Meat eating," "Salt," "Standard bread," "Alcohol," "Open-air treatment," "Hygienic clothing," and many other homely topics are the foundations on which the gay superstructure is raised. We have grown very fond of Dr. Fernie and his entertaining books, and are very grateful to the house of Wright & Sons for discovering him.

**Emergencies of General Practice.** By Percy Sargent, M.B., B.C., and Alfred E. Russell, M.D., B.S. Second Edition. Pp. xi, 454. London: Oxford Medical Publications. [N.D.].—The object with which this book has been written—to bring together "in a concise form for easy reference certain acute and urgent conditions which require prompt recognition and treatment"—has been well attained. The practitioner consulting its pages may rely upon finding practical methods of diagnosis and treatment for all the medical and surgical emergencies he is likely to meet with succinctly described. In this second edition a chapter on the Emergencies of Insanity, by Dr. Percy Smith, has been added, and other chapters have been almost wholly re-written or re-arranged, and the whole work carefully revised.

**Lateral Curvature of the Spine and Flat-foot.** By J. S. Kellett Smith, F.R.C.S. Pp. xii, 137. Bristol: John Wright & Sons Ltd. 1911.—This is a useful little book which deals with the nature of scoliosis and flat-foot and their treatment. The treatment of the extreme cases of scoliosis is not considered, but the treatment of the lesser degrees by various forms of exercises is fully described, and there are numerous and very helpful illustrations. Exercises, both active and passive, are discussed for the treatment of flat-foot, and directions are given for special construction of boots to overcome the deformity. This part of the book is also well illustrated. The book must not be regarded as a complete account of either scoliosis or flat-foot, for the methods of dealing with the worst forms of flat-foot are not referred to, but it seems to us a useful guide to treatment of the less severe forms both of scoliosis and flat-foot.

**The Bradshaw Lecture, December, 1911.** By R. Clement Lucas, B.S., M.B., F.R.C.S. Pp. 50. London: Adlard and Son. 1912.—The subject of this lecture on some points in heredity sounds the parting knell of the word *diathesis*, and gives some interesting illustrations on the Mendelian and Weismannian views on various questions in eugenics. The discovery of the fact that exposure to the X-rays destroys the reproductive function of the generative organs should be of great use in dealing with the problems of the feeble-minded and the survival of the fittest.

**Clinical Methods.** By Robert Hutchison, M.D., and Harry Rainy, M.D. Fifth Edition. London: Cassell and Co. Limited. 1912.—To the casual observer this volume, which now reaches its fifth edition, appears small and easily compassed. However, the memories of a misspent youth teach otherwise. The reviewer has the liveliest recollection of his struggles with the book at the outset of a clinical clerkship, which has now faded into the historic past. In fact, criticism of such a work seems to savour of impiety. The book has become an abiding institution, hallowed by long usage, and no student can afford to be without it during those woeful months when he is wrestling with the elements of physical diagnosis. There is such an enormous mass of information crammed into 600 pages that the reader will find it well to use it as a work of reference after he has gone straight through it once. A certain amount of the subject-matter might perhaps have been left out to make room for more recent methods. There is, for instance, nothing said about the use of the X-rays in diagnosis, and we think the existence of such methods as cystoscopy and bronchoscopy should at least be mentioned. The section which deals with pulse tracings might be made more concise and at the same time more modern. In bringing their book up to date in this fifth edition the authors have invoked the help of several distinguished colleagues, and we trust that this may be taken as evidence of a resolve to keep the book alive and modern by constant revision. Compact, comprehensive, and "level-headed," this volume is indispensable, and not likely to be superseded in the affections of the student.

**The Educational Treatment of Stammering Children.** By Thomas J. T. McHattie, M.D. Pp. 18. London: J. and A. Churchill. 1911.—In this pamphlet, issued by the Medical Officers of Schools Association, Dr. McHattie is an advocate of the psychotherapeutic treatment of stammering, but he would also combine with this the use of deep breathing exercises and lessons in proper voice production. His advice as to the best methods of restoring the self-confidence which is so lacking in the stammerer will well repay study. Some of the most valuable suggestions made in the discussion before the Medical Officers of Schools Association are those of Mr. Cortlandt MacMahon, who dealt with the teaching of classes of stammering children. The institution of centres for this purpose in our large cities is much needed.

**The Therapy of Syphilis.** By Dr. PAUL MÜLZER, Translated by A. NEWBOLD. Pp. xv, 248. London: Rebman Ltd. [N.D.]—This account of the administration of the arsenic compounds, and particularly dioxydiamidoarsenobenzol ("606"), was written in 1910, and the English translation appears somewhat belated. Many observations have been made and published since 1910 which have tended to modify our views of this drug, and to shake our faith in it as the "Therapia sterilisans magna." The book belongs to the period of early enthusiasms, when the possibility of a single injection of "606" curing syphilis was still not disproved. It is well written, well translated, and excellently printed; but the drug has not fulfilled the expectations expressed by the author. For this he must not be blamed. "We do but learn to-day what our better advanced judgments will unteach us to-morrow." Dr. Paul Mulzer's book has helped our judgment to advance.

**The Care of Infants and Young Children.** By H. DINGWALL FORDYCE, M.D. Pp. viii, 158. Edinburgh: E. & S. Livingstone. 1911.—This book deals with the baby in health and disease, and is intended by the author as a guide for mothers, nurses, health visitors and students of medicine. We think that it should be most useful for practitioners also, for it contains many commonsense points in the diet, hygiene, etc., of the child which it is quite impossible to glean from ordinary text-books, and can only be obtained by close observation of the ways and peculiarities of children. In a book of the scope of this one it is only natural that one should find statements with which one cannot agree. The author's condemnation of barley water in the diet of a child seems rather drastic, seeing its frequent use in children's hospitals; also the advice that the child should not be taken out for some weeks after birth seems an unnecessary precaution. The chapter on peculiarities of disease and disorders of digestion in children are particularly good, and his plea for health visitors will be endorsed by many who have seen the same baby again and again in the hospital as a result of the mother not understanding the most elementary principles of feeding. We can heartily recommend the book to those interested in the care of infants and young children.

**Treatment of Tuberculosis and Lupus with Allyl Sulphide.** By WM. C. MINCHIN, M.D. Pp. xi, 88. London: Baillière,

Tindall & Cox. 1912.—Allyl sulphide is the essential oil of garlic, and it has figured in most of the old herbals as a useful remedy, whether taken internally or externally applied, for affections of the chest, as a diuretic, and also as a prophylactic against plague and poisoned wounds including the venom of snakes and dog-bites. Several papers have appeared in recent times concerned with its antiseptic properties, as also with its rapid absorption and excretion by the body. It is the object of this brochure to direct attention to its specificity in tuberculosis, and to suggest methods by which this forgotten herb may be administered with advantage. If it is true, as Dr. Minchin asserts, that "operations for advanced tuberculous disease in accessible situations, *e.g.* amputations of the leg, foot, arm, hand, castration and such-like" are rendered unnecessary by the use of garlic applied as a cataplasm or inhaled from a mask, who is there but would submit to all the evils of the ghetto rather than be dismembered? While if "its peculiar penetrating power and volatile nature makes inhalation quite effective in pulmonary tuberculosis" and in laryngeal phthisis, the "uttering of such a deal of stinking breath" would be forgiven. The writer claims that lupus will yield to applications of the juice, and holds that this treatment has no rivals in the successful management of such cases. These are big claims that he puts forward, and they are capable of proof. If the profession after trial agree with those who have written to Dr. Minchin testifying to the good results that they have obtained or witnessed, it will be under a great obligation to the writer of this little book for reintroducing a cheap, though nasty, cure for the great white plague in its protean forms.

**Dental Anæsthetics.** By Wilfred E. Alderson, M.D., M.S. Pp. viii. 100. Bristol: John Wright & Sons Ltd. 1911.—Although the various branches of medicine have long since been divided up again and again into sub-divisions of specialization, it is questionable whether the title suggested by this book serves a very useful purpose. The phenomena of respiratory anæsthesia is just the same whether it be for dental extraction or removal of ingrowing toe-nail. We think the book, therefore, would have had a wider scope had it been styled "A handbook of anæsthetics for medical and dental students." But while criticising the title of the work, we must congratulate the writer on the lucidity and style of the subject-matter, as well as the arrangement of chapters and the concise way of dealing with anæsthetic administration in its various forms. In glancing through the pages of this little volume just one error—possibly a printer's—strikes the eye. On page 19 the tabulation at the foot of the page should be lettered not numbered, as the last line indicates. We think, in conclusion, that it is just one of those handbooks that every medical as well as dental student ought to have, and its price brings it well within the range of even a modest purse.

**The Prevention of Dental Caries and Oral Sepsis.** By H. P. Pickerill, M.D., M.D.S., L.D.S. Pp. xvi, 308. London: Baillière, Tindall and Cox. 1912.—This is practically the Cartwright Prize Essay of the Royal College of Surgeons for 1909–10. The author was a distinguished student of Birmingham University, and is now the Director of the Dental School at Otago University. It is the record of most original work, leading to most original conclusions. It is not too much to say that if the author's conclusions are correct, and at present we fail to see flaws in the reasoning, we must modify our views on many points. "Modify" is too weak a word, we must start afresh. The abolition of dental caries seems not merely on the horizon, but is apparently in one's grasp. It is a most brilliant essay, and we intend to read it again at leisure.

**Diseases of the Skin.** By Sir Malcolm Morris, K.C.V.O. Fifth Edition, Revised by the Author, with the assistance of S. Ernest Dore, M.D., M.R.C.P. Pp. xv, 762. London: Cassell & Co. 1911.—The five editions through which Sir Malcolm Morris's book has passed since 1893 are sufficient proof of its value and popularity. The gradual addition of new illustrations with successive editions has greatly improved the work. In the treatment of syphilis due consideration is given to the newer methods of arsenical injections and to the serviceability and limitations of the Wassermann test. Ringworm and fungus diseases are dealt with afresh in the light of modern research. Radium-therapy, serum-therapy, vaccine-therapy, and the use of carbon dioxide snow are some of the new subjects introduced. More diseases are recognised as having microbic origins than formerly. In fact, such changes have come into the book that we begin to question whether it is wise to continue bringing out new editions rather than to scrap the old hull, and launch a new design. Many another excellent text-book has suffered from the multiplying of editions. The printing and publishing are of the usual high quality that we look for from the "Belle Sauvage" yard.

**Index-Catalogue of the Library of the Surgeon-General's Office, United States Army.** Second Series. Vol. XVI. Skinko—Stysanus. Pp. 882. Washington, 1911.—The present volume contains 9,890 author titles, 3,892 subject titles of separate books and pamphlets, and 24,135 titles of articles in periodicals. Under "Spinal" and "Spine" there are no less than 123 pages of closely-printed references, and 189 pages are similarly devoted to "Stomach." Such statements show the incalculable worth of such a work to those interested in medical literature, and provide an eloquent testimony to the painstaking care with which the compilers have carried out their arduous task.

